# Extracellular Vesicles as Regulators of the Extracellular Matrix

**DOI:** 10.3390/bioengineering10020136

**Published:** 2023-01-19

**Authors:** Neil J. Patel, Anisa Ashraf, Eun Ji Chung

**Affiliations:** 1 Department of Biomedical Engineering, University of Southern California, Los Angeles, CA 90089, USA; 2 Division of Vascular Surgery and Endovascular Therapy, Department of Surgery, Keck School of Medicine, University of Southern California, Los Angeles, CA 90033, USA; 3 Mork Family Department of Chemical Engineering and Materials Science, University of Southern California, Los Angeles, CA 90089, USA; 4 Eli and Edythe Broad Center for Regenerative Medicine and Stem Cell Research, Keck School of Medicine, University of Southern California, Los Angeles, CA 90033, USA; 5 Division of Nephrology and Hypertension, Department of Medicine, Keck School of Medicine, University of Southern California, Los Angeles, CA 90033, USA; 6 Norris Comprehensive Cancer Center, Keck School of Medicine, University of Southern California, Los Angeles, CA 90033, USA

**Keywords:** extracellular vesicles, extracellular matrix, tissue repair, calcification, tumor microenvironment

## Abstract

Extracellular vesicles (EVs) are small membrane-bound vesicles secreted into the extracellular space by all cell types. EVs transfer their cargo which includes nucleic acids, proteins, and lipids to facilitate cell-to-cell communication. As EVs are released and move from parent to recipient cell, EVs interact with the extracellular matrix (ECM) which acts as a physical scaffold for the organization and function of cells. Recent work has shown that EVs can modulate and act as regulators of the ECM. This review will first discuss EV biogenesis and the mechanism by which EVs are transported through the ECM. Additionally, we discuss how EVs contribute as structural components of the matrix and as components that aid in the degradation of the ECM. Lastly, the role of EVs in influencing recipient cells to remodel the ECM in both pathological and therapeutic contexts is examined.

## 1. Introduction

Extracellular vesicles (EVs) are released into the extracellular environment by all cell types and function as mediators of cell-to-cell communication [1]. EVs contain cargo including lipids, nucleic acids, and proteins that are reflective of their parent cell phenotype [2]. As such, there has been significant interest in utilizing EVs derived from healthy cell sources as endogenous nanotherapeutics [3,4] and applying EVs to enhance angiogenesis, inhibit fibrosis and apoptosis, regulate the immune microenvironment, and modulate the extracellular matrix (ECM) in the context of promoting tissue regeneration [5,6,7,8,9]. 

When EVs are released from parent cells into the extracellular space, they may first interact with the surrounding ECM. The ECM is the physical scaffolding for cellular components of tissues and actively participates in cell growth, movement, and differentiation. The major components of the ECM include collagens, laminins, fibronectin, and proteoglycans that are organized into a physically crosslinked network via non-covalent interactions. The ECM interacts with cells through cell adhesion molecules (e.g., integrins, cadherins, and other transmembrane proteoglycans) which enable cells to migrate across the matrix. Importantly, cells can modify the matrix by depositing ECM components or degrading the ECM by secreting matrix-degrading enzymes such as matrix metalloproteases (MMPs). The mechanical and physical properties of the ECM have been demonstrated to be integral to the differentiation, migration, and maintenance of cells within the ECM. These proprieties include (1) mesh size, which is the distance between two crosslinks within a matrix, (2) stiffness, which is the extent to which the matrix resists deformation when stress is applied, and (3) the viscoelastic behavior, or the ability of the matrix to exhibit both viscous and elastic characteristics upon deformation [10]. Together, the components of the ECM and the mechanical characteristics of the matrix are integral to the health and function of the resident cells [11,12,13,14]. As such, EVs released by both healthy and pathological cells can act as mediators of the ECM either through direct EV–ECM interactions or by influencing cell–ECM interactions.

For the receiving cell, EVs also transport through the ECM of the target tissue to reach the recipient cells and deliver their cargo to impart a cellular response. Transport of EVs through the ECM can be a passive, diffusive process dependent on the stiffness and viscoelastic characteristics of the ECM and the deformability of the EV itself [15,16]. EVs can become structural components of the matrix, either as initiators of calcification or as bioactive signaling agents anchored to the matrix, or can modulate the ECM indirectly by inducing recipient cells to promote synthesis or degradation of the ECM [17,18,19,20,21,22]. 

Given its unstudied but significant role, this review will discuss both the direct and indirect influence of EVs on the ECM. First, the biogenesis and characteristics of EVs and their transport through the ECM will be delineated. Additionally, the role of EVs in modulating the ECM in cancer progression, as structural components during bone/endochondral and vascular calcification, and as ECM-bound bioactive signaling agents will be addressed. Furthermore, how EVs also indirectly modulate the ECM will be explored in both pathological and therapeutic contexts.

## 2. EV Biology

### 2.1. EV Biogenesis

**Figure 1:**
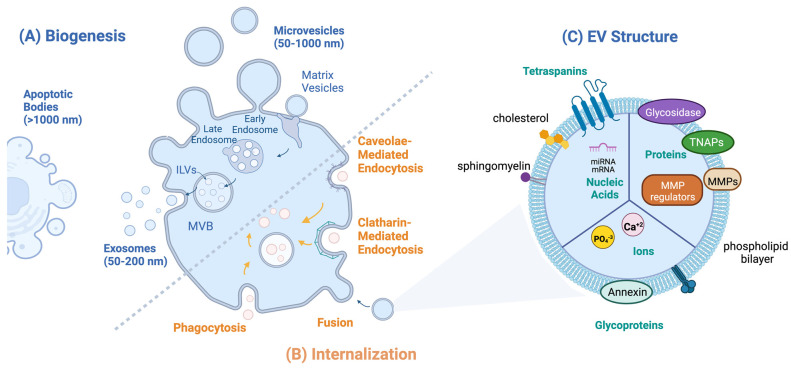
EV biology: (**A**) Routes of EV biogenesis: exosomes are formed from multivesicular bodies (MVB); apoptotic bodies are formed from cells undergoing programmed cell death; microvesicles bud off the donor cell plasma membrane and include matrix vesicles. (**B**) Routes of EV internalization include caveolae-mediated endocytosis, clathrin-mediated endocytosis, micropinocytosis, and phagocytosis, which are internalized via endosomes, and EV fusion with the plasma membrane. (**C**) EV structure: internal cargo includes nucleic acids, proteins, and ions; surface molecules include glycoproteins, tetraspanins, matrix metalloproteases (MMPs), and TNAPs.

EV biogenesis includes vesicle formation, cargo loading, and secretion. Exosome (50–200 nm) biogenesis begins with an early sorting endosome which then matures into a late sorting endosome and finally into a multivesicular body (MVB, Figure 1A). MVB formation is marked by the invagination of the late endosomal membrane forming intraluminal vesicles (ILVs) within the MVB. ILVs are released into the extracellular environment when the MVB merges with the cell membrane and once excreted, they are referred to as exosomes [23]. Exosomes are commonly identified by expression of the membrane-bound tetraspanin marker CD63 and syntenin-1 [24]. Exosome cargo loading is regulated by the endosomal sorting complex required for the transport (ESCRT) pathway, which is comprised of four protein complexes denoted ESCRT-0 through ESCRT-III. ESCRT-0 recruits ubiquitinated protein to the endosomal membrane after which ESCRT-I clusters the cargo and complexes with ESCRT-II [25]. The protein cargo is then sequestered into the endosome and ESCRT-II initiates ILV invagination. Lastly, ESCRT-III de-ubiquinates the protein cargo and finalizes vesicle budding to form ILVs. Additionally, RAB GTPases are associated with exosome formation and support exosome secretion by directing MVBs to the plasma membrane for fusion [26]. 

Beyond exosomes, microvesicles range in size from 50 to 1000 nm and are released by blebbing of the plasma membrane. Thus, they express markers of the originating cell plasma membrane as well as tetraspanins CD9 and CD81 [27,28]. In addition to surface markers, exosome and microvesicle membranes are comprised of cholesterols, sphingomyelins, and phospholipids [29]. Additionally, apoptotic bodies are large EVs (>1000 nm) released when cells undergo programmed cell death [30]. While apoptotic bodies participate in key processes in cell death, such as the removal of cell contents and delivering information from dying cells to phagocytic cells, the majority of EV biology research has been directed towards small EVs including exosomes and microvesicles which range from 50 to 200 nm in size.

### 2.2. EV Uptake

Since exosomes and microvesicles participate in cell-to-cell communication via the transfer of encapsulated messenger RNA (mRNA), microRNAs, small nuclear RNA, long non-coding RNAs, and cytosolic and membrane-associated proteins, they are often uptaken by cells to deliver their cargo [31]. Once released into the extracellular environment, multiple energy-dependent pathways have been proposed by which EVs are internalized by the target cell including clatharin-mediated endocytosis (CME), caveolin-dependent endocytosis (CDE), phagocytosis, and membrane fusion (Figure 1B) [28]. Accordingly, previous studies show that inhibition of the CME pathway by the drug chlorpromazine results in a significant decrease in EV uptake [32]. Similarly, suppressing the cavelin-1 protein, which is necessary for the CDE pathway, resulted in decreased EV endocytosis [31]. Special cases for EV uptake include phagocytic cells and within the tumor microenvironment in which EVs are uptaken primarily by phagocytosis [33]. Additionally, EV-cell membrane fusion as an entry mechanism has also been observed in acidic tumor microenvironments [34].

## 3. EV Transport through the ECM

In order to participate in cell-to-cell communications, EVs must traverse the ECM from the parent cell to the recipient cell. Recent studies regarding stress relaxation of the ECM by Lenzini et al. [15,16] showed that the mechanical properties of the ECM and EVs allow EV diffusion through the extracellular space, despite the larger exosome and microvesicle diameter (50–200 nm) compared to the mesh size of the ECM (~50 nm) [15]. The authors demonstrated approximately 50% of the EVs loaded within decellularized ECM of lung tissue were released after 24 h. By using crosslinked alginate hydrogels with tunable viscoelastic properties, EVs in stiff physically crosslinked alginate hydrogels were found to have significantly higher diffusion coefficients compared to both soft viscoelastic and elastic hydrogels, indicating that the ECM undergoing stress relaxation leads to increased EV diffusion through the matrix. This was true across EVs from a variety of cell sources, implying that the mechanical properties of the matrix play a role in EV transport. In addition, depleting the membrane water channel protein aquaporin-1 on the EV surface increased the stiffness of the EV and reduced the diffusion coefficient by approximately threefold. This indicates that water permeation enables EVs to become more deformable and thus, more easily able to diffuse through the matrix [35].

## 4. Direct Influence of EVs on the ECM

EVs have been reported to directly interact with the ECM and can contribute as both a physical and bioactive structural component of the matrix. In addition, EVs can directly associate with the ECM and actively degrade the matrix with their surface-associated enzymes [5,16,36,37,38,39]. In each case, EVs play an integral role in the evolution of the ECM in both physiological and pathological states (Table 1). 

### 4.1. EV-Mediated Calcification

#### 4.1.1. Bone Formation and Endochondral Calcification

**Figure 2:**
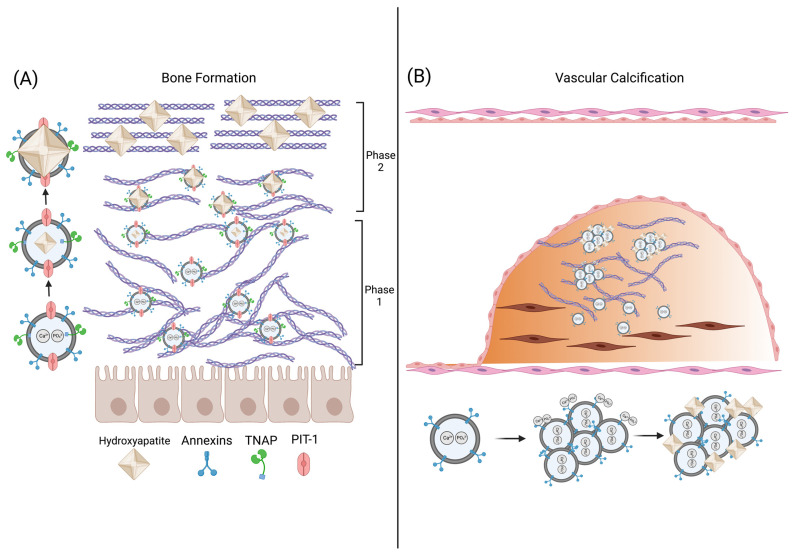
EV-mediated calcification of bone and the vasculature. (**A**) During phase 1 of mineralization during physiological bone formation, osteoblasts release matrix vesicles (MVs) which transport calcium and phosphate ions into the vesicle forming hydroxyapatite (HA) crystals. During phase 2 of mineralization, the HA crystals penetrate the MV and orient themselves with the ECM matrix, furthering crystal growth. (**B**) During vascular calcification, osteogenic-VSMC release EVs that aggregate and anchor to collagen within the ECM. Hydroxyapatite nucleation occurs on the surface of the EV aggregates.

Mineralization of the ECM is a physiological process during bone formation and endochondral calcification while it is pathological during vascular calcification [40]. The ECM architecture during calcification undergoes a dramatic evolution from a non-crystalline network comprised mainly of type 1 collagen fibrils to a crystalline matrix that is able to support high load and stress [41]. Matrix vesicles (MVs, 100–300 nm) are a subtype of microvesicle that are essential to the mineralization of the ECM. MVs released into the ECM from osteoblast and chondrocytes initiate the formation of hydroxyapatite (Ca_10_(PO_4_)_6_(OH)_2_) crystals and the transformation of the ECM from an entirely organic architecture to a combination of both an organic and inorganic (i.e., mineralized) structure. During the first phase of mineralization, MVs sequester Ca^2+^ ions through various calcium-binding molecules concentrated on the MV surface which include the calcium binding proteins annexins II, V, and IV and the membrane phospholipid phosphatidylserine (PS) (Figure 1C and Figure 2A) [42,43,44]. Simultaneously, intra- and extravesicular concentrations of PO_4_^3−^ ions are increased via the following: (1) MV membrane-bound tissue non-specific alkaline phosphate (TNAP), (2) the conversion of ATP to ADP via ATPases, (3) and Pit-1, a sodium-dependent phosphate transporter [44,45,46]. The resultant increased intravesicular Ca^2+^ and PO_4_^3−^ concentrations leads to precipitation of hydroxyapatite crystals within the MV. 

During the second phase of mineralization, hydroxyapatite crystals become large enough to break through the MV into the ECM and continue mineralization of the ECM along the length of type 1 collagen fibrils (Figure 2A). Of note, MV-mediated calcification occurs not only during endochondral calcification during development but also in the constant cycle of bone resorption and formation during adulthood. Thus, MVs are integral in the evolution of the ECM from a viscoelastic network to a fully crystalline structure in physiological calcification processes.

#### 4.1.2. Vascular Calcification 

Vascular calcification is the mineralization of ECM in blood vessels and is present in diseases such as atherosclerosis, diabetes mellitus, and chronic kidney disease [47]. Vascular calcification is initiated with the transdifferentiation of VSMCs into an osteoblast-like phenotype. Under physiological conditions, VSMCs release EVs containing calcification inhibitors such as Matrix Gla protein (MGP) and fetuin-A to maintain tissue homeostasis. However, under pathogenic conditions caused by chronic inflammation or aberrant mineral metabolism, osteoblast-like VSMCs release EVs resembling MVs during bone formation [48]. These EVs can destabilize the ECM of the blood vessel by calcifying distinct regions of the vessel wall, which can lead to thrombosis or rupture of the vessel [49].

The mechanism of EV-mediated vascular calcification differs from that of MV-mediated bone or cartilage mineralization. In calcifying conditions, VSMC-derived EVs are enriched in phosphatidylserine and contain annexins I, II, V, and VI similar to MVs. However, unlike MVs, increased extracellular Ca^2+^ concentration does not lead to an increase in TNAP activity in VSMC-EVs, indicating calcification nucleation is independent of TNAP [50]. Rogers et al. showed that annexin I is enriched in VSMC-derived EVs released during ectopic vascular calcification and facilitates the formation of EV aggregates within collagen fibrils of the ECM. These EV aggregates form mineralization nucleation sites on the surface of the EV which then grow in the presence of increased extracellular Ca^2+^ and PO_4_^3−^ (Figure 2B). Importantly, the knockdown of annexin-I in osteoblast-like VSMCs inhibited the formation of calcification indicating that annexin-I-mediated EV aggregation is necessary for vascular calcification [51]. Thus, unlike bone or cartilage in which mineralization occurs within the MV, vascular calcification is mediated by the surface phospholipids and annexins of EV aggregates localized in the ECM. 

### 4.2. Matrix-Bound Vesicles Are Integral to the Bioactive Properties of the ECM

In addition to acting as structural components during calcification, EVs embedded within the matrix are an integral part of the bioactive properties of the ECM. Recently discovered is a novel subtype of EVs anchored within the ECM called matrix-bound vesicles (MBV) which act as key biological signaling agents within the ECM [52]. Interestingly, MBVs do not contain any of the common exosome or microvesicle markers CD63, CD9, CD81, or HSP70 suggesting a different biogenesis pathway. Additionally, MBVs have tissue-specific characteristics with distinct lipid, RNA, and protein profiles indicating MBVs are an integral part of the bioactive signaling composition of the ECM and have a function in regulating tissue homeostasis [53,54].

Merwe et al. demonstrated that MBV derived from the ECM of the urinary bladder of healthy rats protected against ischemia-induced injury of retinal ganglion cells (RGC) [55]. As decellularized urinary bladder ECM (UB-ECM) had previously been shown to promote ganglion cell axon growth in coculture with microglia and astrocytes, the authors hypothesized that MBVs were a key biological agent anchored within the UB-ECM that were responsible for the increased cell growth [56]. In vitro treatment of a coculture of microglia, astrocytes, and RGC with UB-ECM-derived EVs suppressed the pro-inflammatory signaling of activated microglia and astrocytes and promoted RGC axon growth. Furthermore, intraocular administration of UB-ECM-derived EVs in an optical ischemia murine model ameliorated hypoxia-induced RGC axon degeneration by approximately 80% compared to the uninjured control. Thus, MBVs are key players responsible for the biological effects, namely promoting cell growth and regulating the immune microenvironment, exerted by the ECM. 

Additionally, MBVs have been shown to be integral to ECM-dependent regulation of the immune microenvironment by modulation of immune cells within the matrix. With regards to rheumatoid arthritis, which is an autoimmune disease characterized by chronic inflammation of synovial joints, UB-ECM-derived EVs were shown to prevent acute and chronic arthritis at an efficacy comparable to the clinical standard [57]. Specifically, UB-ECM-derived EVs administered intravenously in an arthritic rat model promoted the transition of pro-inflammatory M1 macrophages to an anti-inflammatory M2 phenotype thereby decreasing inflammation in synovial joints and limiting adverse joint remodeling. Taken together, these studies underscore the indispensable function MBVs have as the bioactive structural component of the ECM. 

### 4.3. EV-Mediated Modulation of the ECM

**Figure 3:**
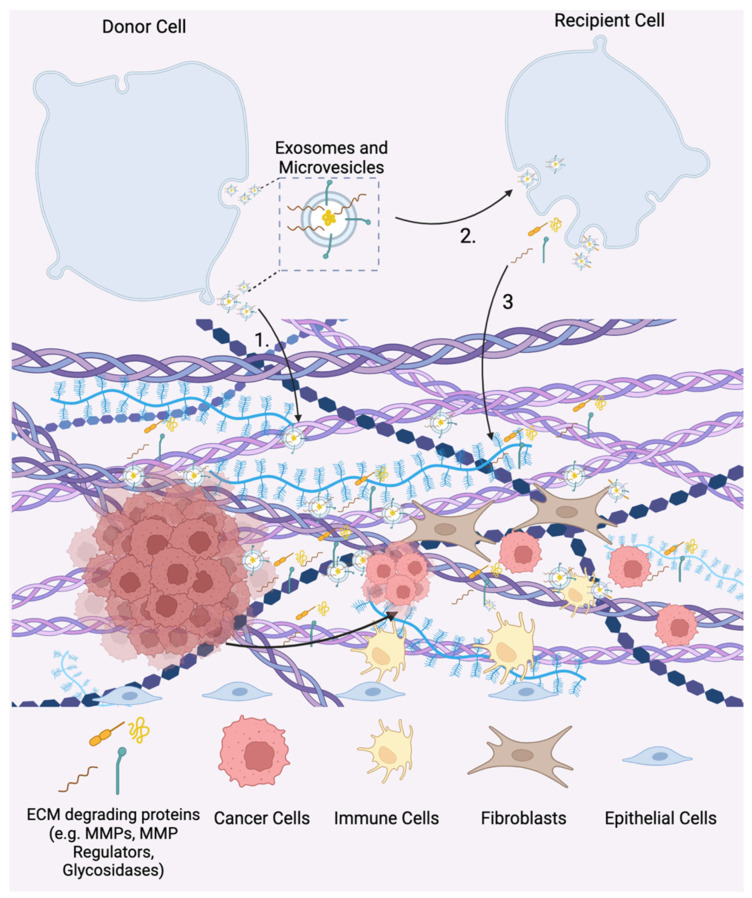
EV-mediated matrix remodeling in tumor progression. 1. Matrix metalloproteases (MMPs) and glycosidases displayed on EV surfaces promote ECM degradation. 2. EVs released from tumor cells are uptaken by recipient cells in both the tumor microenvironment and the pre-metastatic niche. Tumor-derived EVs promote the release of ECM-degrading proteins (e.g., MMPs and MMP regulators) and EVs in recipient cells. 3. Free and EV-associated ECM degrading proteins promote tumor cell invasion, metastasis, and the development of the pre-metastatic niche.

In contrast to calcification in which EVs aid in the structural development of the ECM, EVs released from cancer cells have been shown to actively degrade the ECM via enzymes such as MMPs and glycosidases present on the EV surface [5,16,36,37,38] (Figure 1C and Figure 3). EVs derived from human G361 melanoma cells and HT-1080 human fibrocarcinoma cells display matrix metalloprotease MT1-MMP (MMP-14), a transmembrane protein that promotes cell migration by degrading ECM components such as collagen, fibronectin, and laminins [58,59]. Similarly, EVs derived from nasopharyngeal cancer (NPC) cells were shown to contain matrix metalloprotease 13 (MMP-13) on the EV surface. Autologous incubation of the MMP-13-expressing EVs with NPC cells in Matrigel led to increased degradation of the matrix compared to both EVs with decreased MMP-13 levels and the untreated control, resulting in increased cell invasion within the matrix [60]. In addition to EVs derived from cancer cells, human vascular endothelial cells (HVECs) also secrete EVs that present MMP-2, MMP-9, and MMP-14 on the membrane surface. Incubating Matrigel with HVEC-EVs resulted in the degradation of the matrix and a threefold increase in HVEC cell migration compared to conditions without EV treatment [61]. As evidenced by these studies, EVs have direct contact and actively degrade the ECM by their surface-bound MMPs, resulting in enhanced cell migration through the matrix. 

In addition to MMPs, EVs degrade the ECM by utilizing surface-bound heparinase, a glycosidase that degrades heparan sulfate proteoglycans (HSPC) [36,62,63,64]. HSPC is a is a proteoglycan, which is comprised of a core glycoprotein with multiple heparan sulfate groups that can sequester and bind signaling molecules including growth factors, chemokines, and cytokines to the matrix [65]. EVs derived from myeloma cells have heparinase bound to HSPC on the EV surface [62,63]. When these myeloma-derived EVs were incubated with ECM deposited by endothelial cells, free heparan sulfate chains were detected in the culture media [63]. Since HSPCs sequester bioactive signaling molecules and anchor them to ECM, EV-mediated cleavage of HSPC can modulate the signaling properties of the ECM. 

EVs have also been shown to contain the surface-bound ECM-modulating enzymes lysyl oxidase like-2 (LOXL2), elastase, and collagenase [66,67]. Specifically, Jong et al. showed that EVs from human endothelial cells grown in hypoxic conditions present LOXL2, which is responsible for collagen crosslinking, on the EV surface [66]. Incubation of LOXL2-EVs within a collagen gel increased crosslinking leading to approximately twofold higher stiffness. Thus, hypoxic endothelial cells produce EVs which directly promote collagen crosslinking in the ECM. Conversely, Genshmer et al. found that EVs from activated neutrophils contained surface-bound elastase and collagenase [67]. Incubation of these activated neutrophil-derived EVs with both elastin and collagen fibrils led to a 2.5-fold increase in the degradation of elastin and collagen compared to the inactivated neutrophil EVs. As such, EV-surface-associated enzymes can directly influence the ECM by modulating the matrix through multiple ECM targets including collagen, fibronectin, elastin, and heparan sulfate proteoglycans.

**Table 1:** Direct EV-ECM effects.

	EV Source	EV Effects on the ECM	Active EV Components	Refs.
EV-mediated calcification				
	Osteoblasts	Promotes calcification of the ECM during bone and endochondral calcification	Annexins II, IV, V, Pit-1, Phosphatidylserine, TNAP	[42,43,44]
	Osteochondrogenic vascular smooth muscle cells	Promotes calcification of the vasculature during vascular calcification	Annexins I, II, IV, V, VI	[50,51]
Bioactive signaling Agents				
	Matrix-bound vesicles	Promotes anti-inflammatory signaling and cell growth in retinol ganglion cells and macrophages	N/A	[52,53,54,55,56,57]
Direct modulation of the ECM				
	G361 melanoma cells	Degrades collagen, fibronectin, and laminin; promotes cell migration	MMP-14	[58]
	HT-1080 fibro carcinoma cells	Degrades collagen, fibronectin, and laminin; promotes cell migration	MMP-14	[59]
	Nasal pharyngeal carcinoma cells	Degrades collagen; promotes cell migration	MMP-13	[60]
	Vascular endothelial cells	Degrades collagen, fibronectin, and laminin; promotes cell migration and angiogenesis	MMP-2,9,13	[61]
	Myeloma cells	Degrades heparin sulfate proteoglycans	Heparinase	[62,63,64]
	Neutrophils	Degrades collagen and elastase	Collagenase and elastase	[66]
	Endothelial cells	Promotes collagen crosslinking	Lysyl oxidase like-2	[67]

## 5. Indirect Influence of EVs on the ECM

While EVs can directly modulate the structural components of the matrix as discussed above, EVs can also indirectly alter the ECM by influencing recipient cells to remodel the ECM. Specifically, EVs released from tumor cells have been shown to direct recipient cells to modulate the matrix to create a favorable tumor niche [36]. In addition, EVs released from mesenchymal stem cells (MSCs) have been found to have therapeutic capability by improving ECM remodeling by fibroblasts and chondrocytes during tissue regeneration which will be further detailed below [52,54,55,57,68] (Table 2).

### 5.1. Tumor-Derived EVs Mediate Cell–ECM Interactions during Tumor Progression

#### 5.1.1. Tumor Cell-Derived EVs Promote ECM Degradation

In Section 4.2, it was discussed that EVs derived from cancer cells have matrix-degrading enzymes present on their surface which promotes cancer cell migration and growth. However, in addition to directly interacting with the ECM, EVs secreted into the tumor microenvironment also contain cargo such as MMPs and MMP regulators that is transferred to recipient cells inducing cell-mediated remodeling of the ECM (Figure 1C). For example, EVs derived from hypoxic nasopharyngeal carcinoma (NPC) cells contain MMP-13 as cargo, and treatment of normoxic NPC with hypoxic NPC-derived EVs resulted in a 2.5-fold increase in cellular expression of MMP-13. Additionally, hypoxic NPC-derived EV treatment of normoxic NPC cells increased cell migration and invasion by approximately 300% and 200%, respectively, compared to the non-treated control. Furthermore, EV treatment of normoxic NPC cells decreased levels of the tumor suppressor protein E-cadherin by approximately 80% compared to the non-treated control, thereby promoting tumor growth [69]. Similarly, EVs derived from aggressive myeloma patient cells, which bound to both RPMI-8226 plasmacytoma lymphocytes and human endothelial cells via surface-associated fibronectin, increased the expression of MMP-9 in the RPMI-8226 cells and promoted endothelial cell invasion. Specifically, treatment with myeloma-derived EVs promoted increased cell spreading and migration of endothelial cells through a Matrigel basement membrane by 1.5-fold, indicating that the EVs promote modulation of the matrix via MMP-9 and reprogram the healthy cells towards a more cancerous phenotype [64].

Tumor-derived EVs also contain MMP regulators such as the glycoprotein extracellular matrix metalloprotease inducer (EMMPRIN) which is a transmembrane protein that stimulates the expression of MMPs by tumor-associated fibroblasts. EVs derived from epithelioid sarcoma cells contain EMMPRIN and treatment of human fibroblasts with sarcoma-derived EVs resulted in an increased expression of MMP-2 by approximately twofold compared to both the non-treated control and EVs without EMMPRIN [70]. Another MMP regulator that has been associated with poor prognosis in multiple cancer types is TIMP-1 [71,72]. Overexpression of TIMP-1 in human lung adenocarcinoma cells increased the expression and loading of the pro-angiogenic and pro-tumorigenic microRNA-210 into EVs. These EVs induced angiogenic tube formation in human epithelial cells and promoted lung adenocarcinoma cell migration in vitro, both processes in which ECM remodeling occurs [73,74]. Overall, these studies indicate that tumor-cell-derived EVs promote recipient cells toward a cancerous phenotype in which ECM degradation is involved.

#### 5.1.2. Tumor-Derived EVs Facilitate Pre-Metastatic Niche Formation

The pre-metastatic niche is a potential location of metastasis that has been primed for invasion by circulating tumor cells. According to the “seed and soil” model, metastasis requires that the “soil” (receiving microenvironment) be ideal for the “seed” (circulating tumor cells) to populate [75]. Primary tumors release EVs which actively inform distant recipient cells to modify the ECM and prime the pre-metastatic niche for tumor cell invasion and establishment of a metastatic site. The work of Lyden and colleagues unraveled the role of EVs in establishing an organotropic pre-metastatic niche [17]. They first described that melanoma-derived EVs in circulation educate bone marrow progenitor cells towards a phenotype that supports tumor growth and metastasis [17,21]. In addition, they demonstrated that integrins on the surface of tumor-derived EVs are responsible for organotropic accumulation of EVs [76]. Once localized to a specific tissue, resident cells uptake these tumor-derived EVs and are induced to remodel the ECM toward the development of a pre-metastatic niche.

The role of MMPs and other matrix-modulating proteins in the development of the pre-metastatic niche is well established, and the function of EVs in regulating the expression of these matrix-modulating proteins is now being explored [77]. Redzic et al. showed that EVs released from breast cancer, leukemia, and pancreatic cancer cell lines contain EMMPRIN [78]. When these cell lines were treated with their respective EVs, MMP-9 and IL-6, which are downstream targets of EMMPRIN, were upregulated across all cell lines. Additionally, the cells expressed a higher level of EMMPRIN and increased their secretion of EMMPRIN-containing EVs.

Furthermore, melanoma-derived EVs have also been shown to facilitate ECM deposition and promote angiogenesis to prime sentinel lymph nodes for tumor metastasis. Specifically, intravenous injection of melanoma-derived EVs into mice led to an increase in mitogen-activated protein kinase 14, laminin, collagen, and urokinase plasminogen activator, or ECM-modulating factors involved in stroma remodeling that enable invasion by circulating tumor cells. Establishing a pre-metastatic niche in sentinel lymph nodes by intravenous administration of melanoma EVs led to increased selective colocalization of circulating melanoma cells with melanoma EVs in the sentinel lymph nodes [79]. As such, tumor-derived EVs can create an organotropic pre-metastatic niche through influencing recipient cells to modulate the ECM.

### 5.2. Therapeutic Potential of EVs in Tissue Regeneration

While EVs released from pathogenic cells are involved in disease progression, EVs from healthy cells have become of interest as next-generation therapeutics. Their low immunogenicity, high biocompatibility, endogenous therapeutic cargo, and ability to efficiently transfer bioactive agents to target cells have made them an attractive therapeutic platform in recent years [80]. While there are no current EV therapeutics in clinical trials specifically related to the ECM, multiple studies have reported the therapeutic ability of EVs to promote cell-dependent modulation of the matrix during tissue regeneration.

**Table 2:** Indirect effect of EVs on the ECM.

Function	EV Source	EV Target	Cell–ECM Interactions	Active EV Components	Refs.
Tumor EVs promote ECM degradation					
	Hypoxic nasal pharyngeal carcinoma cells	Normoxic nasal pharyngeal carcinoma cells	Increases cell expression of MMP-13, decreases expression of E-cadherin, promotes cell migration and invasion	MMP-13	[69]
	Human myeloma cells	RPMI-8226 plasmacytoma lymphocytes and human endothelial cells	Increases MMP-9 expression, promotes cell invasion, and migration	MMP-9, fibronectin	[64]
	Epithelioid sarcoma cells	Fibroblasts	Increases expression of MMP-2	EMMPRIN	[70]
	Human lung adenocarcinoma cells	Human epithelial cells and lung adenocarcinoma cells	Induces angiogenic tube formation and promotes cell migration	TIMP and miR-210	[73,74]
Tumor EVs create a pre-metastatic niche					
	Human melanoma cells	Bone marrow progenitor cells	Promotes tumor growth and metastasis	N/A	[17]
	Breast cancer MCF-7 cells	Human fibroblasts	Increases expression of MMP-9 and IL-6	EMMPRIN	[78]
	Monocytic leukemia U937 cells	Human fibroblasts	Increases expression of MMP-9 and IL-6	EMMPRIN	[78]
	Pancreatic cancer L3.6pL cells	Human fibroblasts	Increases expression of MMP-9 and IL-6	EMMPRIN	[78]
	Melanoma cells	Sentinel lymph nodes	Increases in mitogen-activated protein kinase 14, laminin, collagen, and urokinase plasminogen activator	N/A	[79]
Mesenchymal stem cell EV-mediated ECM repair					
	Adipose tissue MSCs	Dermal fibroblasts	Increases the ratio of type 3 collagen to type 1 collagen and the ratio of MMP-3 to TIMP-1	N/A	[81,82]
	Adipose tissue MSCs	Chondrocytes	Promotes type 2 collagen synthesis and inhibits the expression of MMP-1, MMP-3, MMP-13, and ADAMTS-5	N/A	[83]
	Bone marrow MSCs	Bone marrow MSCs	Promotes cell adhesion and motility	Hyaluronan	[84]

#### Mesenchymal Stem Cell EV-Mediated ECM Repair

EVs derived from mesenchymal stem cells (MSC) have been shown to participate in restoring ECM during tissue regeneration. For example, EVs derived from adipose tissue MSC (AT-MSC) decreased scar formation and aided in wound healing in an in vivo model. Specifically, AT-MSC EVs were administered intravenously to a murine model with a dorsal wound. AT-MSC-EV treatment increased the ratio of type 3 collagen to type 1 collagen and MMP-3 to TIMP-1 in dermal fibroblasts, thereby aiding in matrix remodeling and limiting scar formation [81]. Interestingly, fibroblasts isolated from women with stress urinary incontinence that had significantly decreased expression of type 1 collagen, treated with AT-MSC EVs had increased expression of type 1 collagen and TIMP-1/3, and downregulation of MMP-1/2 [82]. Increased deposition of collagen along with inhibition and decreased expression of MMPs led to the restoration of ECM stiffness that is lost during stress urinary incontinence. Woo et al. demonstrated that AT-MSC EVs could also attenuate the progression of osteoarthritis, which is a chronic degenerative disease of articular cartilage. In vitro, AT-MSC EV treatment promoted chondrocyte migration and proliferation while simultaneously promoting type 2 collagen synthesis and inhibiting the expression of MMP-1, MMP-3, MMP-13, and ADAMTS-5. Additionally, intra-articular injections of AT-MSC EVs inhibited osteoarthritis progression in a murine model by protecting the cartilage matrix both by increased collagen deposition and inhibition of ECM-degrading proteases [83]. In addition to MMPs, human bone marrow (BM) MSC secretes hyaluronan-coated EVs. Hyaluronan (HA) is a glycosaminoglycan that acts as a critical bioactive factor within the ECM that plays roles in cell adhesion, motility, proliferation, and differentiation. HA-coated EVs were shown to be secreted from BM-MSC and anchored onto the ECM providing bioactive signaling cues within the matrix [84]. Taken together, these studies suggest that stem-cell-derived EVs have promise as therapeutics for tissue regeneration via modulation of the ECM.

## 6. Conclusions

During pathological processes such as tumor progression, EVs function via both the direct (i.e., EV-surface-associated enzymes) and indirect mechanisms (i.e., influencing recipient cells) to aid in ECM degradation, tumor progression, and the development of the pre-metastatic niche. Similarly, in physiological processes such as bone formation and tissue regeneration, EVs from osteoblasts and MSCs can directly evolve the structure of the ECM through calcification or by directing recipient cells to remodel the ECM during tissue repair, respectively. More knowledge regarding EV–ECM interactions during pathological processes such as cancer will allow for new therapeutic and diagnostic approaches targeting EV-mediated tumor progression and metastases. Similarly, characterizing the effect of EVs on the ECM during physiological processes such as tissue regeneration presents the opportunity to create novel or augment current therapeutic approaches while overcoming translational limitations in tissue repair.

## Data Availability

No new data was created or analyzed. Data sharing is not applicable to this article.
